# Tuber mustard *BjuFIP* gene negatively regulates plant sensitivity to abscisic acid

**DOI:** 10.1038/s41598-025-29074-3

**Published:** 2025-12-29

**Authors:** Zhaoming Cai, Ningfei Shi, Yijie Chang, Siqin Liu, Diandong Wang, Chunhong Cheng

**Affiliations:** College of Life Science and Technology, Yangtze Normal University, Chongqing, 408100 People’s Republic of China

**Keywords:** Tuber mustard (*Brassica juncea* var. *tumida*), Abscisic acid, F-box protein Interacted with BjuPYL3 (BjuFIP), Abiotic stress, Plant sciences, Plant development, Plant stress responses

## Abstract

Tuber mustard (*Brassica juncea* var. *tumida*) is an economically important vegetable crop, with its tumorous stem serving as the primary raw material for Fuling mustard production. However, multiple abiotic stress during growth often leads to significant yield losses. As a vital hormone for plant stress adaptation, abscisic acid (ABA) plays essential roles in stress responses. However, despite the agricultural importance of tuber mustard, no studies have yet reported on ABA related functions in this crop. In this study, we identified the F-box protein gene *BjuFIP* as a key regulator in ABA signaling. *BjuFIP* was ubiquitously expressed across all tissues and significantly upregulated by ABA treatment. Histochemical analysis of *Pro*_*BjuFIP*_*::GUS* transgenic *Arabidopsis* seedlings further confirmed ABA-induced *BjuFIP* expression. Functional characterization revealed that *Arabidopsis* overexpressing *BjuFIP* exhibited an ABA-insensitive phenotype, suggesting that *BjuFIP* negatively regulates ABA signaling in tuber mustard. Furthermore, protein–protein interaction assays, including yeast two-hybrid and bimolecular fluorescence complementation, demonstrated that BjuFIP physically interacts with BjuASK1, a core component of the SCF ubiquitin ligase complex, and BjuPYL3, an ABA receptor. These findings suggest that BjuFIP might modulate the ABA signaling pathway through regulation of BjuPYL3. These findings not only advance our understanding of ABA signaling in tuber mustard but also offer valuable genetic targets for breeding stress-tolerant varieties with stable yields.

**Supplementary Information:**

The online version contains supplementary material available at 10.1038/s41598-025-29074-3.

## Introduction

The ubiquitin-mediated protein degradation system is a highly efficient and selective regulatory mechanism that is widely conserved in eukaryotes. This system plays crucial roles in diverse cellular processes including cell cycle regulation, differentiation, programmed cell death, and stress responses^1–3^. The SCF complex consists of three core subunits: SKP1 (S-phase kinase-associated protein 1), CUL1 (Cullin 1 scaffold protein), and an F-box protein^4^. The F-box protein functions as the substrate recognition subunit, determining target specificity^5,6^. SCF-type E3 ubiquitin ligases play crucial roles in plant stress responses^7,8^. *Arabidopsis* plants overexpressing AtPP2-B11 exhibit reduced drought tolerance but enhanced salt stress resistance^9,10^. In wheat, TaFBA1 as an F-box protein, showed significant upregulation under various stress conditions, and transgenic tobacco plants overexpressing *TaFBA1* demonstrate markedly improved tolerance to heat, ABA, and drought stresses^11,12^.

ABA serves as a crucial phytohormone mediating plant adaptation to abiotic stress^13^. Environmental stresses such as low temperature and drought trigger substantial ABA accumulation in plants, subsequently activating ABA-responsive gene expression. The ABA signaling pathway, which governs these stress responses, comprises four core components: ABA receptors (PYR1/PYL/RCAR family), protein phosphatase 2C (PP2C), SNF1-related protein kinase 2 (SnRK2), and bZIP transcription factors that regulate downstream ABA-responsive genes. Under normal growth conditions, when endogenous ABA levels are low, PP2C phosphatases maintain pathway inactivation by dephosphorylating and inhibiting SnRK2 kinases, preventing induction of ABA-responsive genes. However, under stress conditions, rapid ABA synthesis occurs, and ABA binding to PYR1/PYL/RCAR receptors relieves PP2C-mediated inhibition of SnRK2 kinases, thereby activating the ABA signaling pathway^14,15^. The PYR1/PYL/RCAR family consists of 14 soluble ABA receptor proteins (PYR1 and PYL1-PYL13)^16–19^. Among these, PYL3 functions as a positive regulator in ABA signaling through multiple mechanisms: direct ABA binding, ABA-dependent interaction with PP2C, and inhibition of PP2C phosphatase activity to modulate plant stress responses^16–20^. For instance, in rice, OsPYL3 expression is strongly induced by various stresses and ABA treatment. *Arabidopsis* plants overexpressing *OsPYL3* exhibit both enhanced ABA sensitivity and improved tolerance to cold and drought stress^21^. In tuber mustard, the PYR1/PYL/RCAR family expands to 25 members. Notably, BjuPYL3 shows particularly high expression in roots and significant induction under low-temperature stress, suggesting its specialized role in cold stress response^22^.

Tuber mustard, an economically important vegetable crop in China, is primarily cultivated for its swollen stem, which serves as the key raw material for Fuling pickles^23^. The botanical classification of this crop was first scientifically established in 1942 by Professors Mian Zeng and Shuxuan Li of Jinling University, who identified it as a distinct variety within the species *Brassica juncea* (Brassicaceae family). This taxonomic designation has been widely recognized by the international botanical community. Throughout its growth cycle, tuber mustard frequently encounters various abiotic stresses including low temperature, salinity, and drought conditions^24–26^. These environmental constraints significantly inhibit stem swelling and leading to substantial yield reductions and considerable economic losses. Despite its agricultural importance, research on stress resistance mechanisms in tuber mustard remains limited. Few resistance genes have been identified to date, and their functional characterization and molecular mechanisms are largely unexplored.

*AtPP2-B11* is a stress-resistant gene that forms the SCF^AtPP2-B11^ complex to regulate plant responses to ABA, drought, and salt stress in *Arabidopsis*^8–10^. In previous study, we cloned two homologous genes of *AtPP2-B11* in tuber mustard (*BjuPP2-B11-1* and *BjuPP2-B11-2*). Quantitative real-time PCR expression analysis revealed that compared to *BjuPP2-B11-1*, *BjuPP2-B11-2* showed significantly stronger induction by ABA, NaCl, and mannitol treatments after 3 h stresses treatment. Based on these findings, we selected the *BjuPP2-B11-2* gene for further functional characterization. In this study, we found that BjuPP2-B11-2 was an F-box protein and could interact with ABA receptor BjuPYL3, leading us to rename *BjuPP2-B11-2* as *BjuFIP* (F-box protein Interacting with BjuPYL3). To investigate whether BjuFIP mediates tuber mustard response to abiotic stress, we analyzed its gene expression pattern and found ubiquitous expression across all tissues with ABA induction. *Arabidopsis* overexpressing *BjuFIP* exhibited an ABA-insensitive phenotype. BiFC assays confirmed the interaction between BjuFIP and BjuPYL3, suggesting that BjuFIP may mediate tuber mustard ABA response through this interaction.

## Materials and methods

### Plant growth conditions and ABA treatment

The *Arabidopsis* wild type (Col-0) and tuber mustard cultivar ‘Yong’an Xiaoye’ used in this study was provided by Yangtze Normal University. Both wild-type *Arabidopsis* (Col-0) and transgenic *BjuFIP*-overexpressing seeds were surface-sterilized with 50% (v/v) sodium hypochlorite solution for 10 min, followed by three rinses with sterile distilled water. The sterilized seeds were then sown on MS basal medium supplemented with vitamins. After stratification at 4 °C for 2 days in darkness to synchronize germination, the plates were transferred to a growth chamber maintained at 22 °C under long-day conditions (16-h light/8-h dark photoperiod).

For ABA sensitivity phenotypic analysis, we prepared 200 mL of MS solid medium, autoclaved it, and allowed it to cool to approximately 60 °C. Under sterile conditions in a laminar flow hood, 2 μL of 50 mM ABA stock solution (final concentration: 0.5 μM) was added, thoroughly mixed, and poured into sterile Petri dishes to prepare solid culture plates. These plates were used for sowing *Arabidopsis* seeds and subsequent phenotypic analysis.

For qPCR analysis, 200 mL of MS solid medium was prepared, autoclaved, and cooled to about 60 °C. Under sterile conditions, 200 μL of 50 mM ABA stock solution (final concentration: 50 μM) was added, mixed well, and poured into Petri dishes to prepare solid culture plates for seedling transfer. Tuber mustard seeds were first sown on MS medium, and after 7 days, the seedlings were transferred onto ABA-containing MS plates (50 μM). Samples were collected at 0, 3, 6, 12, and 24 h after ABA treatment, flash-frozen in liquid nitrogen, and processed for RNA extraction, cDNA synthesis, and subsequent qPCR analysis.

For GUS staining, 100 μL of 50 mM ABA stock solution was added to 100 mL of sterile water (final concentration: 50 μM). After mixing, 20 mL of the solution was added to Petri dishes lined with sterile filter paper. Wild-type and transgenic *Arabidopsis* samples at different developmental stages, including imbibed seeds, 1-day-old, 5-day-old, and 7-day-old seedlings, stems and rosette leaves of 3-week-old plants, inflorescences, siliques, and immature seeds were placed on the filter paper and incubated for 3 h before histochemical GUS staining.

### Transgenic homozygous line screening and identification

To generate transgenic plants overexpressing *BjuFIP* under the control of the cauliflower mosaic virus 35S promoter, we amplified the coding sequence of *BjuFIP* by PCR and cloned it into the pTF101vector containing the Barselectable marker gene. *Arabidopsis* transformation was performed using the floral dip method with *Agrobacterium tumefaciensstrain* EHA105. After seed collection, putative transformants were selected on MS medium supplemented with Basta (final concentration: 5 μL of 10% Basta per 100 mL MS medium). Transgenic lines with single insertion events were identified based on a 3:1 segregation ratio, and homozygous lines were subsequently isolated for gene expression analysis and phenotypic characterization.

### Phenotypic analysis assay

For phenotypic analysis of *BjuFI*P-overexpressing seedlings under ABA treatment, seeds were plated on MS medium supplemented with or without 0.5 μM ABA (three replicate plates per treatment, with 30 seedlings per line per plate). Following stratification at 4 °C for three days, the plates were transferred to a growth chamber under controlled environmental conditions. Germination and greening were assessed at specified time points using the following criteria: elongated radicles (a radicle length of more than half of the seed was used as the standard for seed germination) and green cotyledons, acting as the criteria for germinated seeds and greening seedlings, were counted at the indicated time points.

### Gene expression analysis

The samples of root, stem, tumorous stem, leaf, flower, inflorescence, pod, and seven-day-old tuber mustard seedlings treated with 50 μM ABA for 0, 3, 6, 12, and 24 h, respectively, were collected for RNA isolation. RNA was isolated using TRIzol reagent. Reverse transcription was then performed using cDNA synthesis Supermix with gDNA remover (TransGen Biotech, Beijing, China). Quantitative real-time PCR was performed using the ABI 7500 Real-Time PCR System. Transcript abundance was normalized against the reference gene *BjuACTIN3*. The experiments were performed three times with three replicates each. The primers are shown in Table S1.

### GUS histochemical analysis

For the GUS histochemical assay, the 2000 bp fragment upstream of the ATG of *BjuFIP* was cloned into the pCAMBIA1391 vector. Different tissues of transgenic plants were either treated or not treated with 50 μM ABA for 3 h. The samples were then incubated in GUS staining buffer containing 5-bromo-4-chloro-3-indolyl-β-D-glucuronic acid overnight at 37 °C. After GUS staining, the samples were cleared with 75% ethanol.

### Yeast two hybrid and BiFC analysis

The coding sequences of *BjuFIP*, *BjuPYL3*, and *BjuASK1* were cloned using specified primers, inserted into the pDONOR207 vector, and then recombined in the Gateway Destination vectors pGBKT7, pGADT7, pEarleyGate201-YN, and pEarleyGate202-YC for the vector construction of BjuFIP-BD, BjuASK1-AD, BjuPYL3-YC, BjuFIP-YN, and BjuASK1-YC. For the yeast two hybrid assay, the plasmids BjuFIP-BD and BjuASK1-AD, AD and BD (negative control), and AD-T and BD-53 (positive control) were transformed into yeast strain AH109. The co-transformed AH109 was cultured in the SD media lacking Leu and Trp or lacking Ade, His, Leu, and Trp. For the BiFC assay, *A. tumefaciens* carrying BjuFIP-YN and *A. tumefaciens* carrying BjuPYL3-YC, *A. tumefaciens* carrying BjuFIP-YN and *A. tumefaciens* carrying BjuASK1-YC, *A. tumefaciens* carrying BjuFIP-YN and *A. tumefaciens* carrying YC, *A. tumefaciens* carrying YN and *A. tumefaciens* carrying BjuASK1-YC, *A. tumefaciens* carrying YN and *A. tumefaciens* carrying BjuPYL3-YC, *A. tumefaciens* carrying YN and *A. tumefaciens* carrying YC were co-infiltrated into tobacco leaves. The YFP signals were observed using a laser scanning microscope 48 h after infiltration. The fluorescent dye DAPI (4′,6-diamidino-2-phenylindole) was used as a chromosome and nuclear stain.

### Bioinformatics analysis

The gene sequences of *AtPP2-B11* were searched in TAIR (http://www.arabidopsis.org/), the protein sequence of AtPP2-B11 was used as the query sequence to blast the homologous protein in tuber mustard using *Brassica* database (http://brassicadb.cn/#/), the advanced parameters value is 1e−5. The protein motifs of AtPP2-B11 and BjuFIP were analyzed by SMART (http://smart.embl-heidelberg.de/) and the CDS and protein sequences alignment was analyzed by ClustalX.

### Statistical analysis

All of the statistically analysis data in Figs. 2, 3 and 4 were analyzed using SigmaPlot 10.0 (Systat Software, Inc., Chicago, IL, USA) with three independent biological replicates. The averages and standard deviations of all results were calculated, and Students’ t-tests were performed to generate *P* values.

## Results

### F-box protein BjuFIP interacts with BjuASK1 in Y2H and BiFC assays

Based on protein sequence homology alignment, two AtPP2-B11 homologous proteins (BjuPP2-B11-1, BjuPP2-B11-2) were identified in tuber mustard. The sequence identity between *BjuPP2-B11-2* (*BjuFIP*) and *AtPP2-B11* was analyzed using ClustalX and found that they had high CDS sequence identity (83.14%) and protein sequence identity (76.36%) (Fig. S1A). *BjuPP2-B11-1*, *BjuPP2-B11-2* and *AtPP2-B11* shared the similar gene structure, and the proteins they encoded all contained an F-box domain at the N-terminus (Fig. S1B and Fig. S1C). In previous study, we found that compared with *BjuPP2-B11-1*, *BjuPP2-B11-2* was significantly induced by ABA, NaCl, and mannitol. Additionally, BjuPP2-B11-2 interacts with the ABA receptor BjuPYL3. Therefore, BjuPP2-B11-2 was renamed BjuFIP (F-box protein Interacting with BjuPYL3) and selected for in-depth investigation.

**Fig. 1:**
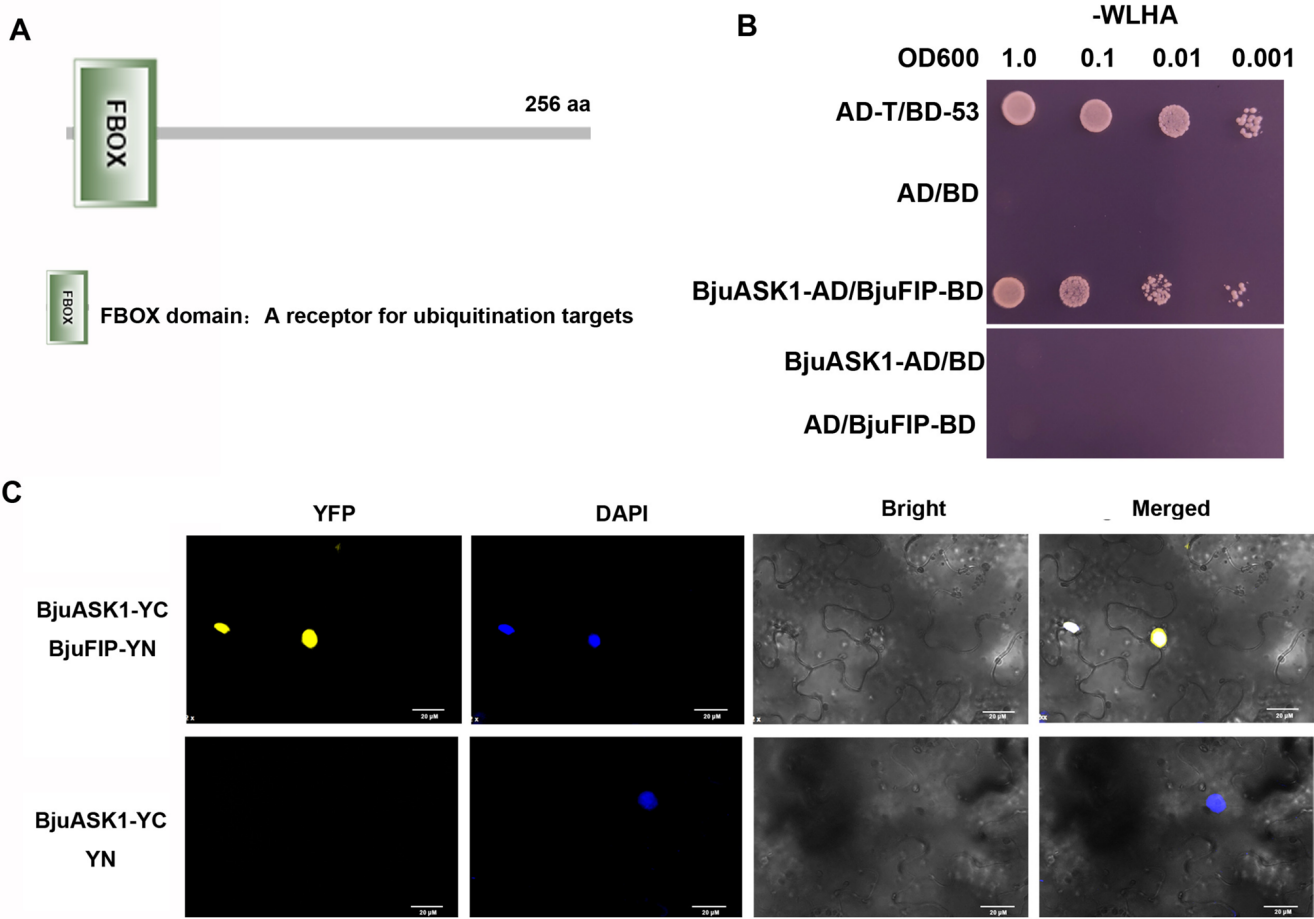
Protein motif analysis of BjuFIP and the interaction analysis of BjuFIP with 3 BjuASK1 in Y-2-H and BiFC assays.4 (**A**) Protein motif analysis of BjuFIP. FBOX domain: a receptor for ubiquitination targets. 5 (**B**) The F-box protein BjuFIP was found to interact with BjuASK1 by Y-2-H growth 6 assay performed on SD media lacking Leu and Trp (-WL) and Ade, His, Leu, and Trp 7 (-WLHA). Saturated cultures were spotted onto SD media lacking Ade, His, Leu, and 8 Trp (-WLHA) at different dilutions (OD600 = 1, 0.1, 0.01, and 0.001). The vectors AD-T 9 and BD-53 were used as positive controls and the emptyvectors AD and BD were used 10 as negative controls. (**C**) The interaction between BjuFIP-YN and BjuASK1-YC in 11 tobacco leaves was analyzed by a BiFC assay. The YFP signal (left), DAPI (second 12 column), bright field image (third column), and merge image (right) are shown.13.

Through protein conserved domain analysis, it was found that BjuFIP contains an F-box domain (Fig. 1A, and Fig. S1C). BjuASK1 (an SKP1 family protein) is a member of the SCF-type E3 ubiquitin ligase complex, responsible for bridging the protein CUL1 and the receptor protein F-box to form an SCF-type E3 ubiquitin ligase complex. To determine whether BjuFIP is a member of the SCF-type E3 ubiquitin ligase, yeast two-hybrid and BiFC experiments were performed to verify the interaction between BjuFIP and BjuASK1. As shown in Fig. 1B, C, there was a strong interaction between BjuFIP and BjuASK1 in yeast and tobacco leaves, indicating that BjuFIP might form the SCF^BjuFIP^ complex, acting as the substrate receptor to mediate the degradation of target proteins (Fig. 1B, C).

### *BjuFIP* is expressed ubiquitously in all tissues and induced by ABA

**Fig. 2:**
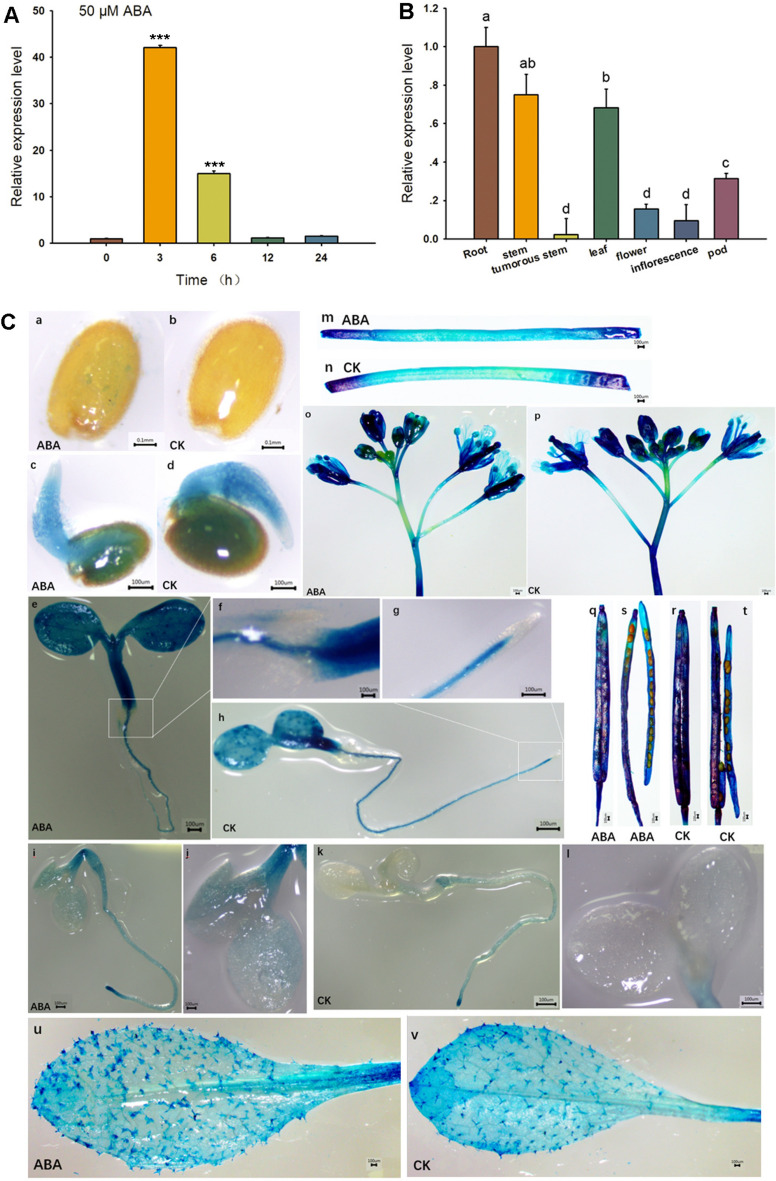
*BjuFIP*is ubiquitously expressed and induced by ABA.14 (**a**) Gene expression pattern of *BjuFIP* was analyzedinseven-day-old tuber mustard 15 seedlingsunder ABA treatment. Seven-day-old tuber mustard seedlings grown on MS 16 media were treated with 50 μMABAat 0, 3, 6, 12, and 24 h. Three independent 17 experiments were performed with similar results, each with three replicates. 18 *BjuACTIN3*was used as the internal control. The statistically significant treatments 19 were marked with “***”(P < 0.001). (**b**) The tissue-specific expression pattern of*BjuFIP*20 was analyzed by qPCR. *BjuACTIN3*was used as the internal control. The different 21 letters indicated significant differences between treatments (P < 0.05). (**c**) GUS staining 22 of *BjuFIP*overexpressing *Arabidopsis*transgenic lines treated with or without 50μM23 ABAfor 3 h. **a** Imbibition seeds treated with 50μMABAfor 3 h. **b** Imbibition seeds 24 treated without ABA.**c** One-day-old imbibed seeds treated with 50μMABAfor 3 h. (**d**) 25 One-day-old imbibed seeds treated without ABA. (**e**) Five-day-old seedlings treated with 26 50μMABAfor 3 h. (**h**) Five-day-old seedlings treated without ABA. (**f**) **and** (**g**) Amplified 27 view of root tip of e and h. (**i**) Seven-day-old seedlings treated with 50μMABAfor 3 h. 28 (**k**) Seven-day-old seedlings treated without ABA. (**j**) **and** (**i**) Amplified view of cotyledon29 of i and k. (**m**) Stems of three-week-old seedlings treated with 50μMABAfor 3 h. (**n**) 30.Stems of three-week-old seedlings treated without ABA. (**u**) Rosette leaves of three-31 week-old seedlings treated with 50μMABAfor 3 h. (**v**) Rosette leaves of three-week-32 old seedlings treatedwithout ABA.**o** Inflorescences, (**q**) Siliques, (**s**) Immature seeds 33 treated with 50μMABAfor 3 h. (**p**) Inflorescences, (**r**) Siliques, (**t**) Immature seeds treated 34 without ABA. CK: Samples of GUS staining assay treated without ABA; ABA: 35 Samples of GUS staining assay treated with 50μMABAfor 3 h.36

The expression profile of *BjuFIP* under ABA treatment was analyzed by qPCR. Seven-day-old tuber mustard seedlings were treated with 50 μM ABA at the indicated time points to measure *BjuFIP* expression levels. The results showed that *BjuFIP* was significantly induced by ABA and reached its highest expression level at 3 h after treatment (Fig. 2A). To study the temporal and spatial expression pattern of *BjuFIP* in different tissues, RNA was extracted from the root, stem (unexpanded), tumorous stem (expanded), leaf, flower, inflorescence, and pod of tuber mustard and qPCR was conducted. The result showed that *BjuFIP* was expressed in all tissues of tuber mustard, with the highest expression in root, stem and leaf, whereas the lowest expression level was in the tumorous stem (Fig. 2B).

To further determine the tissue- and cell-specific pattern of *BjuFIP* expression and response to ABA, we generated transgenic *Arabidopsis* plants expressing GUS under the control of the native promoter of *BjuFIP*. The histochemical staining results showed that *BjuFIP* was expressed in all tissues and was responsive to ABA in the cotyledons and roots of seven-day-old seedlings (Fig. 2C). In the absence of ABA, *BjuFIP* was highly expressed in seeds after germination for one day, but not in radicle root tips and imbibed seeds (Fig. 2C–b and Fig. 2C–d). A high expression level of *BjuFIP* was detected in the cotyledons and shoots of five-day-old seedlings (Fig. 2C–h and Fig. 2C–g). Regardless of ABA treatment, *BjuFIP* was not expressed in the root tips of 5five-day-old seedlings; however, a high expression level was detected in the root tips of seven-day-old seedlings (Fig. 2C–f, Fig. 2C–g, Fig. 2C–i, and Fig. 2C–k). High transcript level of *BjuFIP* was observed in the rosette leaves, trychomes in leaves, pods, and inflorescences regardless of ABA treatment (Fig. 2C–u, Fig. 2C–v, Fig. 2C–o, Fig. 2C–p, Fig. 2C–q and Fig. 2C–r). Furthermore, *BjuFIP* was induced by ABA specifically in the cotyledons and roots of seven-day-old seedlings (Fig. 2C-i, Fig. 2C–j, Fig. 2C–k, Fig. 2C–l).

### *BjuFIP* is a negative regulator of ABA signaling

**Fig. 3:**
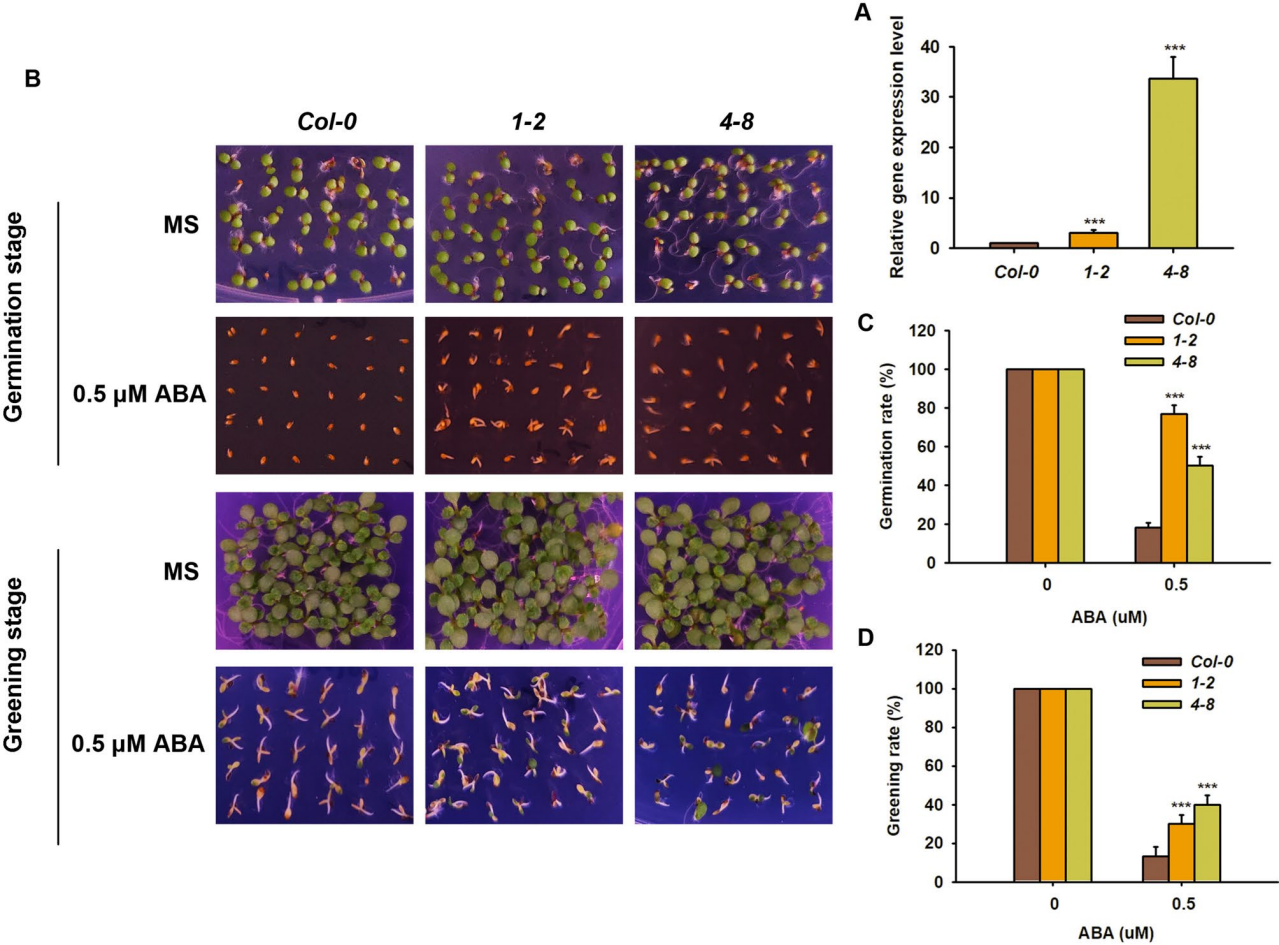
BjuFIP is a negative regulator in the ABA signaling pathway.37 (**A**) Gene expression levels of *Col-0*and *BjuFIP* over expressing transgenic lines*.* 38 *AtACTIN8*was used as the internal control. The statistically significant treatments were 39 marked with “***”(P < 0.001).(**B**) Phenotype analysis of *Col-0*and *BjuFIP*40 overexpressing transgenic lines*1-2*and *4-8*with 0.5μMABAtreatment and control 41 (MS, no treatment). (**C**) The germination rates and (**D**) Greening rates for *Col-0*and 42 *BjuFIP*overexpressing transgenic lines*1-2*and *4-8*with 0.5μMABAtreatment and 43 control (MS, no treatment). Three independent experiments were performed with 44 similar results, with three replicates each. The statistically significant treatments were 45 marked with “***”(P < 0.001).46.

To verify the biological function of *BjuFIP*, transgenic *Arabidopsis* plants overexpressing *BjuFIP* were generated. Putative transgenic lines *1–2* and *4–8* were screened, and qPCR analysis was performed to assess *BjuFIP* expression levels. The results showed that *BjuFIP* expression was significantly higher in transgenic lines *1–2* and *4–8* compared to Col-0, confirming that these lines were indeed *BjuFIP*-overexpressing transgenic plants (Fig. 3A). Next, we analyzed the germination and greening phenotypes of Col-0, *1–2*, and *4–8* with and without ABA treatment. In the absence of ABA, seed germination and cotyledon greening in *BjuFIP*-overexpressing transgenic plants were normal, showing no significant difference from the wild-type Col-0 (Fig. 3B). However, under 0.5 μM ABA treatment, the germination and greening rates of lines *1–2* and *4–8* were significantly higher than those of Col-0. These results indicate that *BjuFIP*-overexpressing transgenic plants exhibit an ABA-insensitive phenotype (Fig. 3B–D).

**Fig. 4:**
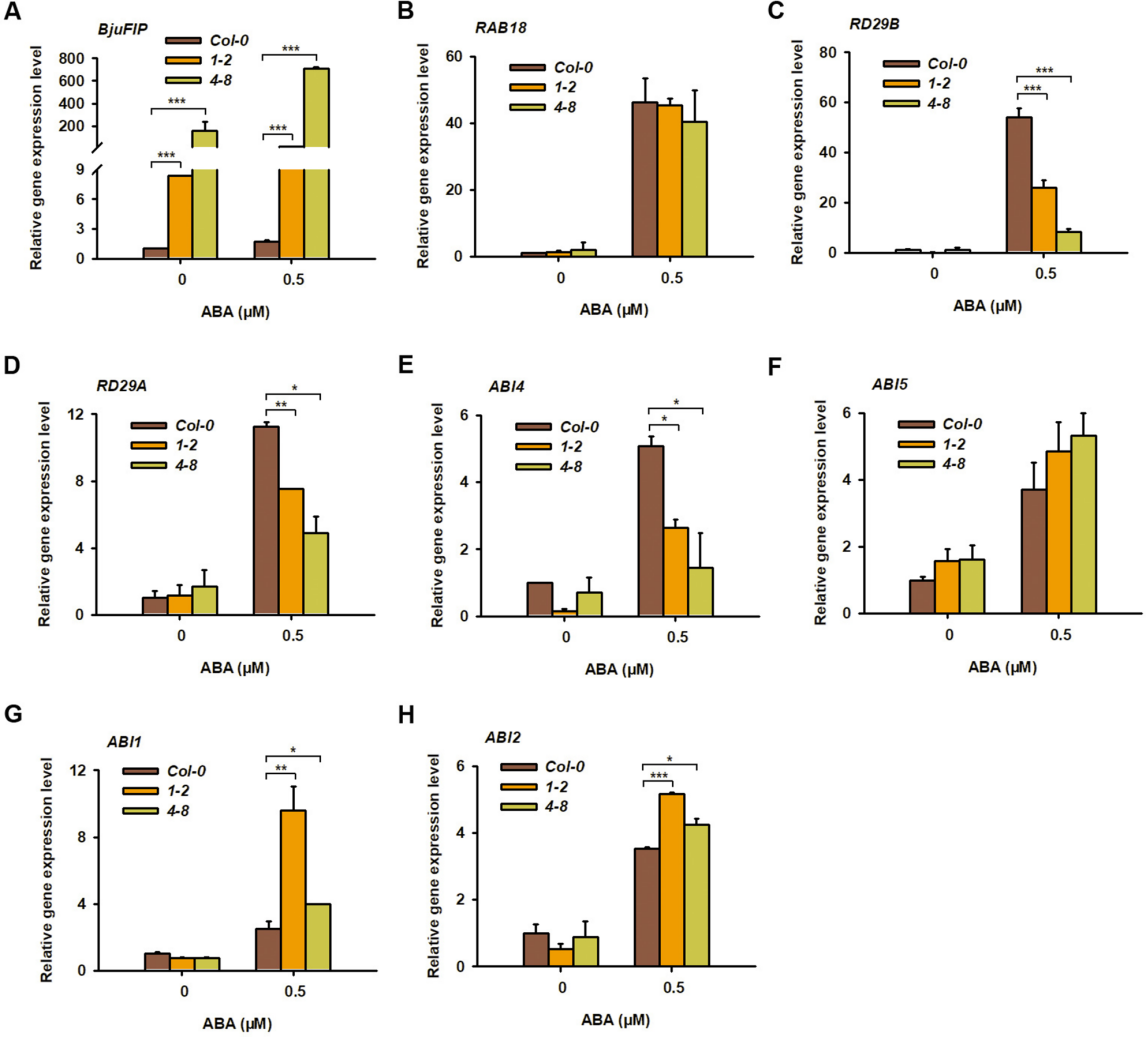
The gene expression levels of ABA responsive genes in *Col-0*and *BjuFIP*47 overexpressing transgenic lines.48 the gene expression level of (**A**) *BjuFIP*, (**B**) *RAB18*, (**C**) *RD29B*, (**D**) *RD29A*, (**E**) *ABI4*, (**F**) 49 *ABI5*,(**G**) *ABI1*, and (**H**) *ABI2*were analyzed in *Col-0*and *BjuFIP*overexpressing 50 transgenic lines*1-2*and *4-8*with 0.5μMABAtreatment and control (MS, no treatment). 51 Three independent experiments were performed with similar results, each with three 52 replicates. *AtACTIN8* was used as the internal control. The statistically significant 53 treatments were marked with “***”(P < 0.001), “**”(0.01 > P > 0.001), and “*”54 (0.01 < P < 0.05).55.

To further demonstrate that *BjuFIP* is a negative regulator of the ABA signaling pathway, we analyzed the expression levels of ABA-responsive genes using qPCR. Seedlings of Col-0 and transgenic lines 1–2 and 4–8 grown on MS medium with or without 0.5 μM ABA were used for the qPCR assay. The results showed that *BjuFIP* was induced by ABA in both Col-0 and the transgenic lines (Fig. 4A). Similarly, the expression levels of *RAB18*, *RD29A*, *RD29B*, *ABI4*, *ABI5*, *ABI1*, and *ABI2* were induced by ABA in Col-0, transgenic lines 1–2, and 4–8 (Fig. 4B–H). Under ABA treatment, the expression levels of *RD29A*, *RD29B*, and *ABI4* were lower in transgenic lines 1–2 and *4–8* than in Col-0, further supporting that *BjuFIP* negatively regulates ABA signaling (Fig. 4C–E).

## ABA receptor BjuPYL3 interacts with F-box protein BjuFIP

**Fig. 5:**
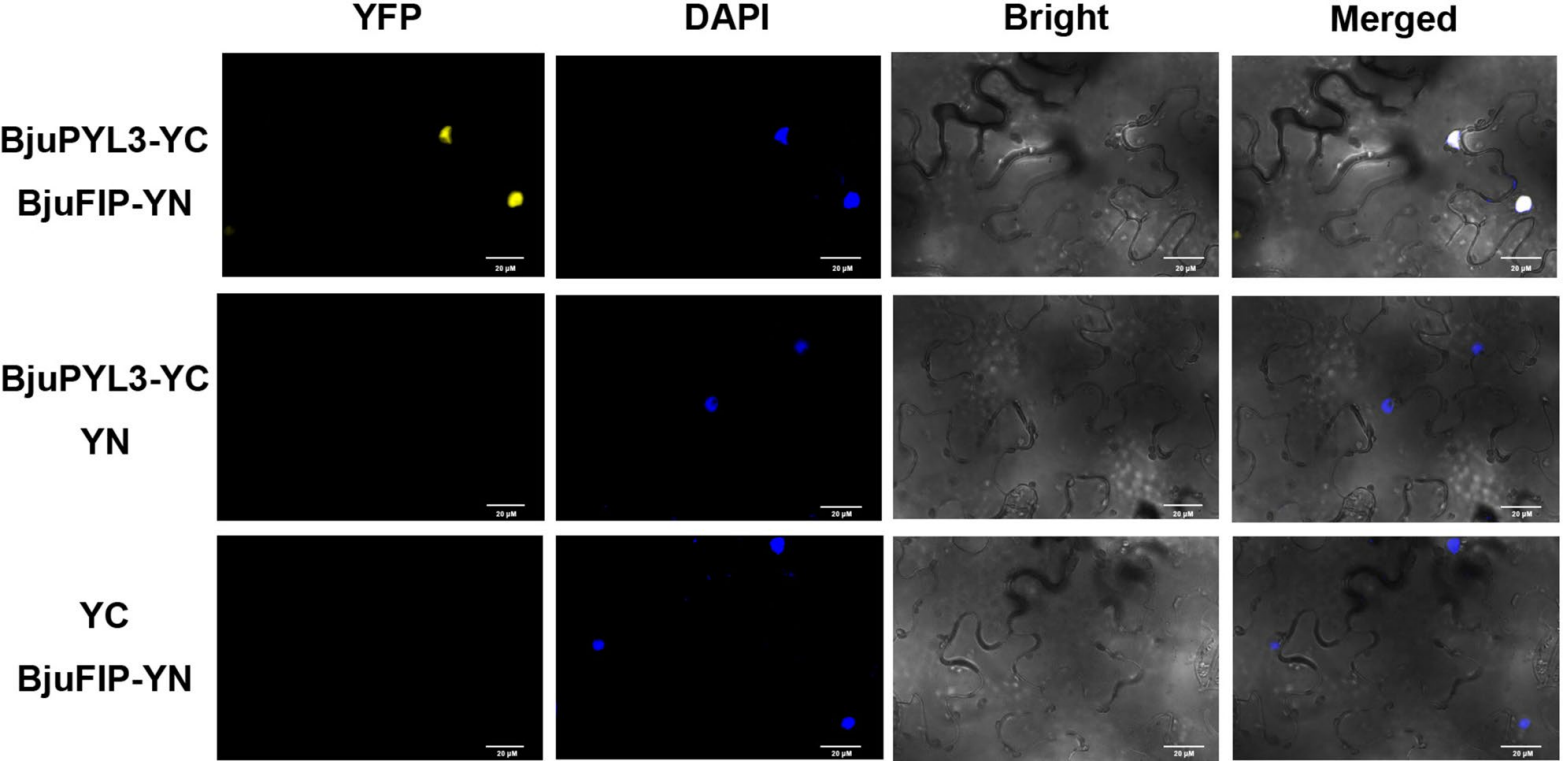
BjuFIP interacts with BjuPYL3 in the BiFC assay.56 the interaction between BjuFIP-YN andBjuPYL3-YC in tobacco leaves was analyzed 57 by a BiFC assay. The YFP signal (left), DAPI (second column), bright field image (third 58 column), and merge image (right) are shown. The BjuFIP-YN/YC and YN/BjuPYL3-59 YC were used as negative controls.60.

To further investigate the mechanism by which BjuFIP regulates tuber mustard response to abiotic stress, we screened for candidate interacting proteins of BjuFIP using yeast two-hybrid (Y2H) libraries, with BjuFIP-BD as the bait protein. An interaction between the ABA receptor BjuPYL3 and BjuFIP was detected in yeast. To confirm this interaction, we performed a bimolecular fluorescence complementation (BiFC) assay. A strong YFP signal was observed in the nucleus after co-expressing BjuFIP-YN and BjuPYL3-YC in tobacco cells for 48 h, whereas no fluorescence signal was detected in negative controls (Fig. 5). PYL3, as an ABA receptor, is a positive regulator in the ABA signaling pathway and plays a role in plant responses to abiotic stress. These results demonstrate that BjuFIP and BjuPYL3 interact in plant cells, suggesting that BjuFIP might modulate tuber mustard response to ABA by affecting BjuPYL3, which requires further verification.

## Discussion

In this study, we present multiple lines of evidence supporting the role of BjuFIP as a key regulator of ABA responses: qPCR and GUS staining demonstrated ABA induction of *BjuFIP* expression (Fig. 2A, C); *BjuFIP*-overexpressing *Arabidopsis* seedlings exhibited ABA-insensitive phenotypes during germination and greening (Fig. 3); The ABA-responsive genes *RD29A*, *RD29B*, and *ABI4* showed lower expression in transgenic lines versus Col-0 under ABA treatment (Fig. 4C–E), indicating BjuFIP played negative regulatory role in ABA signaling.

ABA is a key phytohormone regulating plant growth, development, and stress responses. PYL3 functions as an ABA receptor and positive regulator of ABA signaling^16–18^. Previous studies have shown that ABA receptors can be degraded via the 26S ubiquitin–proteasome pathway. For instance, DDA1- and RSL1-dependent degradation of PYL8 and PYL4 occurs through this pathway^27,28^. However, research on PYL3 has primarily focused on transcriptional regulation, with no reports on its protein stability. Our Y2H and BiFC assays demonstrated that BjuFIP can interact with both BjuASK1 (a key component of the SCF complex) and the ABA receptor BjuPYL3 (Figs. 1 and 5), suggest that BjuFIP might regulate BjuPYL3 stability through the SCF-type ubiquitination pathway. Alternatively, the functional mechanism underlying the BjuFIP-BjuPYL3 interaction might involve competitive binding to ABA receptors to suppress downstream ABA signaling cascades. Previous studies have demonstrated that ABT, another ABA-negative regulatory protein in *Arabidopsis*, can interact with both ABA receptor PYR1/PYL/RCAR and protein phosphatase PP2C proteins. This interaction interferes with PYR1/PYL4-ABI1/ABI2 complex formation and impairs the inhibition of ABI1/ABI2 by ABA-bound PYR1/PYL4, thereby attenuating ABA signaling^15^. Further investigation is required to determine which of these mechanisms mediates how the BjuFIP-BjuPYL3 interaction regulates ABA sensitivity in tuber mustard.

When the content of ABA in plants increases, ABA receptors undergo a conformational change and bind to PP2C (Protein Phosphatase 2C), thereby releasing the kinase activity of SnRK2s. This further leads to the activation of downstream transcription factors in the ABA signaling pathway, such as ABI4 and ABI5. Through transcriptional activation, these factors induce the expression of ABA-responsive genes including *RAB18*, *RD29A*, and *RD29B*. As key components in the activation of the ABA signaling pathway, *ABI4*, *ABI5*, *RAB18*, *RD29A*, and *RD29B* are typically induced to express by ABA^29^. Consistent with this, in the present study, we also found that these genes were significantly upregulated in the wild-type *Arabidopsis* treated with 50 μM ABA (Fig. 4). Previous studies have shown that *Arabidopsis* EAR1 is a negative regulator in the ABA signaling pathway, and the expression levels of *RD29A*, *RD29B*, *RAB18*, *ABF4*, and *KIN1* were lower in EAR1-overexpressing seedlings than in the wild type^30^. Similarly, in the transgenic plants overexpressing BjuFIP, the ABA-induced expression of *ABI4*, *RD29A*, and *RD29B* were significantly inhibited (Fig. 4). However, the ABA-induced expression of *RAB18* and *ABI5* could not be significantly suppressed by BjuFIP. These results suggest that BjuFIP may regulate plant sensitivity to ABA by affecting the expression of *ABI4*, *RD29A*, and *RD29B*. As for the functions of ABI4, RD29A, and RD29B in regulating the response of tuber mustard to ABA, they deserve further investigation.

## Supplementary Information

Supplementary Information 1.

Supplementary Information 2.

Supplementary Information 3.

## Data Availability

Data is provided within the manuscript or supplementary information files.
