# Supplementary figures and images for: Tuber mustard BjuFIP gene negatively regulates plant sensitivity to abscisic acid
Source: Sci Rep. 2025 Dec 29;15:44770. doi: 10.1038/s41598-025-29074-3 (PMC12749184; doi:10.1038/s41598-025-29074-3)

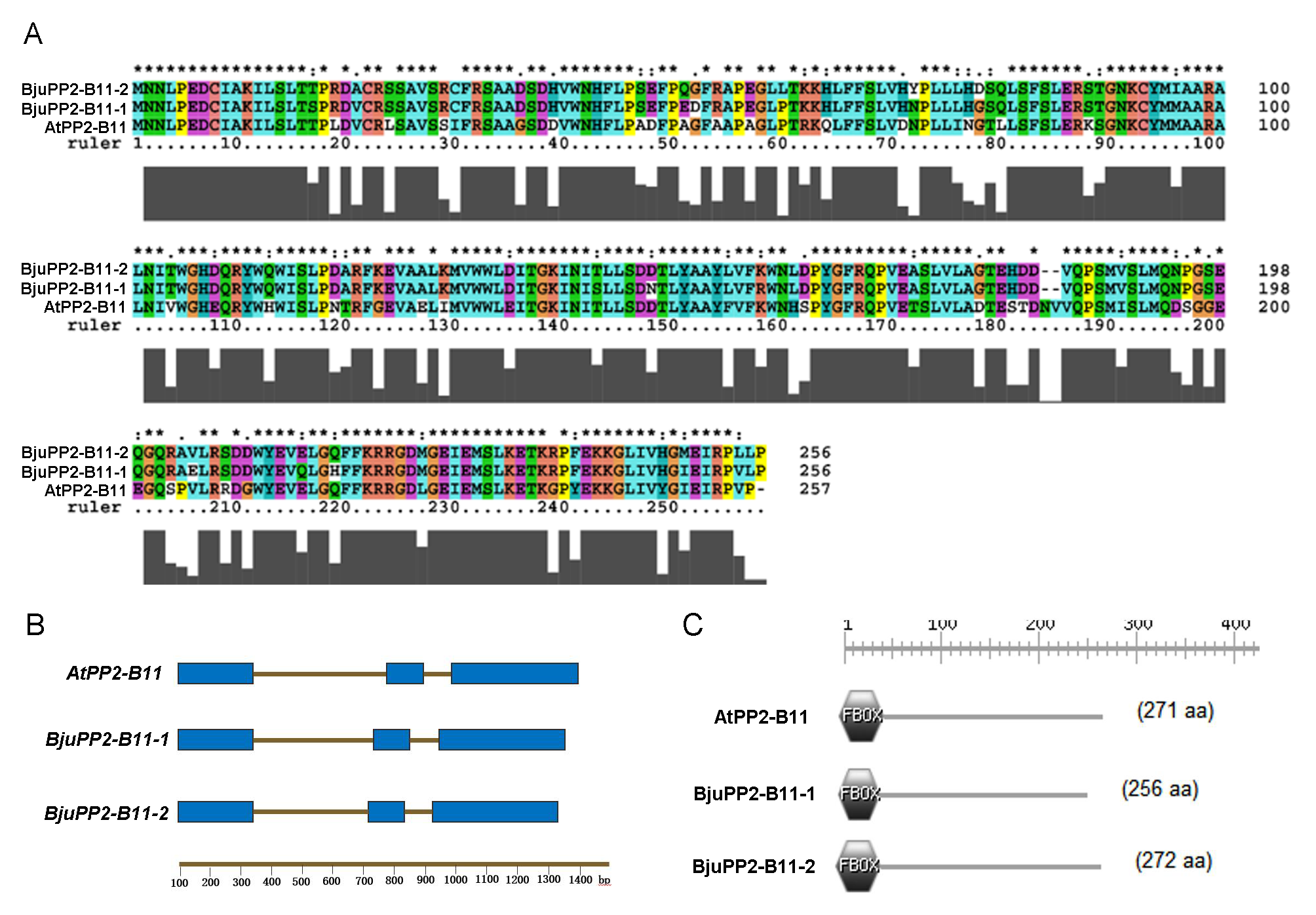

Supplement: Supplementary file 1 — Supplementary Information 1. [file 41598_2025_29074_MOESM1_ESM.tif]
