# Supplementary material for: Tuber mustard BjuFIP gene negatively regulates plant sensitivity to abscisic acid
Source: Sci Rep. 2025 Dec 29;15:44770. doi: 10.1038/s41598-025-29074-3 (PMC12749184; doi:10.1038/s41598-025-29074-3)
Supplement: Supplementary file 2 — Supplementary Information 2. [file 41598_2025_29074_MOESM2_ESM.pdf]

Fig. S1: The sequence alignment and protein domain analysis of BjuFIP and AtPP2-B11.

A The CDS sequence alignment of *BjuFIP* and *AtPP2-B11*. B The protein sequence alignment of BjuFIP and AtPP2-B11. The red box shows the protein sequence of FBOX domain. **C** The protein motif analysis of AtPP2-B11. **D** The protein motif analysis of BjuFIP.

Table S1: The primers used in this study.
