# Supplementary material for: Tuber mustard BjuFIP gene negatively regulates plant sensitivity to abscisic acid
Source: Sci Rep. 2025 Dec 29;15:44770. doi: 10.1038/s41598-025-29074-3 (PMC12749184; doi:10.1038/s41598-025-29074-3)
Supplement: Supplementary file 3 — Supplementary Information 3. [file 41598_2025_29074_MOESM3_ESM.pdf]

Table S1 The primers used in this study

Primer Name	Sequence	Purpose
BjuPYL3-F-gateway	GGGGACAAGTTTGTACAAAAAAGCAGGCTTCATGAATCCTAATCTACCTTCTAGCC	
BjuPYL3-R-gateway	GGGGACCACTTTGTACAAGAAAGCTGGGTCAGTTGGAGAAGCCATGGAAACGG	
BjuFIP-F-gateway	GGGGACAAGTTTGTACAAAAAAGCAGGCTTCATGAATAATCTGCCAGAGGACTGC	Y-2-H and BiFC assays
BjuFIP-R-gateway	GGGGACCACTTTGTACAAGAAAGCTGGGTCGGGCAGTACTGGCCTAATCTC	
BjuASK1-F-gateway	GGGGACAAGTTTGTACAAAAAAGCAGGCTTCATGTCGACGAAGAAGATCGTGTTGAAGAG	
BjuASK1-R-gateway	GGGGACCACTTTGTACAAGAAAGCTGGGTCTTCAAAAGCCCATTGGTTCTCCCTG	
ProBjuFIP-F	GTCGACATGTAAATGAAATATGCGAC	GUS staining assay
ProBjuFIP-R	GAATTCTGCTTTGCTTTTTAGTTTTTAC	
BjuFIP-CDS-F	ATGAATAATCTGCCAGAGGACTGC	Phenotypic analysis
BjuFIP-CDS-R	GGGCAGTACTGGCCTAATCTC	
qRT-BjuFIP-F	CTTCTTCTCCCTCGTCCATAAC	
qRT-BjuFIP-R	CTAGCAGCCATCATGTAGCA	
qRT-RAB18-F	GGCTTGGGAGGAATGCTTCA	
qRT-RAB18-R	CGCTTGAGCTTGACCAGACT	
qRT-RD29B-F	GAATCAAAAGCTGGGATGGA	
qRT-RD29B-R	TGCTCTGTGTAGGTGCTTGG	
qRT-RD29A-F	GGAAGTGAAAGGAGGAGGAGGAA	
qRT-RD29A-R	CACCACCAAACCAGCCAGATG	
qRT-ABI4-F	GGGCAGGAACAAGGAGGAAGTG	
qRT-ABI4-R	TCTCCTCCAAAAGGCCAAATGGT	Gene expression levels analysis
qRT-ABI5-F	ATGATCAAGAACCGCGAGTCTGC	
qRT-ABI5-R	CGGTTGTGCCCTTGACTTCAAAC	
qRT-ABI1-F	AGAGTGTGCCTTTGTATGGTTTTA	
qRT-ABI1-R	CATCCTCTCTCTACAATAGTTCGCT	
qRT-ABI2-F	GATGGAAGATTCTGTCTCAACGATT	
qRT-ABI2-R	GTTTCTCCTTCACTATCTCCTCCG	
qRT-AtACTIN8-F	TCAGCACTTTCCAGCAGATG	
qRT-AtACTIN8-R	ATGCCTGGACCTGCTTCAT	
qRT-BjuActin3-F	GGCTACTCTTTCACCACGAC	
qRT-BjuActin3-R	GGATACCAGCATTCTCCATAC	
